# Specular reflection intensity modulated by grazing-incidence diffraction in a wide angular range

**DOI:** 10.1107/S2053273318008963

**Published:** 2018-09-01

**Authors:** K. V. Nikolaev, I. A. Makhotkin, S. N. Yakunin, R. W. E. van de Kruijs, M. A. Chuev, F. Bijkerk

**Affiliations:** a MESA+ Institute for Nanotechnology, University of Twente, The Netherlands; b NRC Kurchatov Institute, Moscow, Russian Federation

**Keywords:** crystal surface, grazing-incidence X-ray diffraction, GID, specular reflection, azimuthal rocking curves

## Abstract

Grazing-incidence X-ray diffraction (GID) is a well known technique for the characterization of crystal surfaces. A theoretical study has been performed of the sensitivity of GID to the structure of a crystal surface and distorted nanometre-thin surface layers. To simulate GID from crystals that have a complex subsurface structure, a matrix formalism of the dynamical diffraction theory has been applied. It has been found that the azimuthal rocking curves of a crystal that has a distorted subsurface, measured over a wide angular range, show asymmetric thickness oscillations with two distinguishable sets of frequencies: one corresponding to the diffraction in the single-crystal subsurface layer and the second corresponding to the diffraction in the single-crystal substrate. Therefore, azimuthal rocking curves allow characterization of the subsurface structure of a single crystal. Furthermore, thickness oscillations induced by evanescent diffraction modulate the specular reflection intensity, showing high-intensity modulations. This will potentially allow implementation of subsurface crystal characterization using, for instance, a laboratory-scale X-ray diffractometer.

## Abstract 2

A theoretical description is given of the novel X-ray diffraction effect in single-crystal structures with a distorted crystal subsurface based on the dynamical theory of diffraction.

## 1. Introduction   

The motivation for the characterization of crystal surfaces can be found in, for example, the development of topological insulators (Ngabonziza *et al.*, 2015 ▸) and spin-injection structures (Aronzon *et al.*, 2008 ▸) in which their properties depend on the crystal subsurface structure. However, the characterization of this crystal subsurface is challenging because it typically requires high-brilliance synchrotron radiation (Robinson, 1986 ▸) that is generally not as readily available as radiation from compact laboratory-scale X-ray source setups.

Theoretical study of the diffraction of evanescent X-rays and its relation to the dispersion surface (Afanas’ev & Melkonyan, 1983 ▸) has shown that diffraction in grazing-incidence geometry is sensitive to the surface structure of the crystals. In particular, grazing incidence ensures a limited penetration depth, and thus the beam mainly irradiates the surface and subsurface of the crystal. Near-surface horizontally propagating X-rays diffract from crystal planes perpendicular to the surface if the angle between the X-ray beam and the crystal planes satisfies the Bragg condition. This effect has been demonstrated experimentally (Cowan *et al.*, 1986 ▸).

However, the intensity of the diffracted beam is very low in grazing-incidence X-ray diffraction (GID), necessitating synchrotron-based experiments. Yet the specular reflection intensity, at grazing incidence, is much higher than that of diffraction. It has been shown by Bushuev & Oreshko (2001 ▸), Bushuev *et al.* (2001 ▸) that specular reflection intensity is modulated by the Bragg peak in GID. The intensity of the specular reflection in the Bragg condition for GID gives information about the structure of the subsurface layer, namely, thickness, amorphization and deformation [for example with use of a static factor (Afanasev *et al.*, 1977 ▸)].

However, the analysis discussed above is limited to the Bragg condition. Yet information about the depth profile of the lateral lattice parameter and the relative lattice orientation is within a range that is far from the GID Bragg condition. In order to obtain the structural parameters of ultra-thin crystal subsurface layers, we here propose to extend the analysed angular range further away from the Bragg condition. In numeric simulations we observed that a GID beam far from the Bragg condition also modulates a specular reflection intensity.

In this article, we present a theoretical study of reflection intensity modulation involving the use of the matrix formalism of the dynamical diffraction theory (Stepanov & Kohler, 1994 ▸). A minor difference in structures of the bulk and the surface of the crystal significantly affects the GID intensity. The phase of the wave diffracted from the surface is slightly different from the phase of the wave diffracted from the bulk crystal substrate. Both these waves interfere, in a similar way to an acoustic beat, creating thickness oscillations with two distinguishable sets of frequencies on the rocking curves far from the Bragg condition.

We have derived approximate expressions for the dispersion surface in order to understand how the parameters of the structure affect specular reflection intensity modulation. In addition to thickness, deformation and amorphization, one can analyse the depth profiles of the lateral lattice parameter and the lattice orientation. Finally, a wider angular range allows us to increase the precision of estimation of these parameters compared with estimates in previous studies.

To study the feasibility of such measurement using low-intensity sources, we have conducted numerical simulation of specular reflection intensity while assuming typical parameters for a laboratory-scale X-ray instrument. Based on the results of this simulation, we conclude that it is feasible to take such a measurement using a laboratory X-ray source.

## 2. Theoretical background: dynamical theory of diffraction   

In this section, we review the matrix formalism (Stepanov *et al.*, 1998 ▸; Caticha, 1993 ▸) of the dynamical diffraction theory (Pinsker, 1978 ▸) that we implement for the numerical simulations in §4. The problem of X-ray diffraction is approximated by the scalar wave equation (Pietsch *et al.*, 2013 ▸): 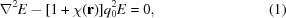
where *E* is the scalar amplitude of the polarized electric wave and 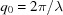 is the wavenumber of the wave in vacuum with wavelength λ. This equation was derived by assuming that diffraction is an elastic scattering process and the magnetic permittivity is equal to unity 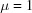. The crystal structure is represented with a dielectric susceptibility 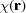 as a function of coordinate 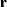.

**Figure 1:**
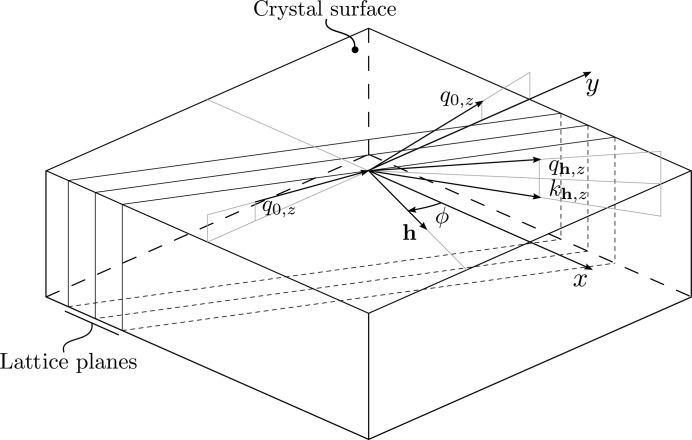
Geometry of the GID azimuthal rocking-curve measurement.

In the GID geometry (see Fig. 1 ▸), one has to consider multiple scattering processes. Therefore, the dynamical theory of diffraction is used for GID. In that theory the solution of the wave equation is in the form of the Bloch wave (Holý & Fewster, 2003 ▸). The matrix formalism of the dynamical diffraction theory (Stepanov *et al.*, 1998 ▸) considers two main scattering processes: reflection and diffraction. Within such a two-beam approximation, the wavefield has the form
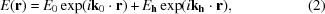
where 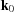 and 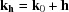 are the wavevectors and 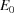 and 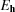 are Fourier components of the electric field corresponding to the transmitted and diffracted waves, respectively, and 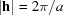 is the reciprocal-lattice vector, perpendicular to the lattice planes with spacing *a*. The dielectric susceptibility χ describes the optical properties of the medium (Born & Wolf, 2000 ▸). Following the two-beam approximation, in a medium with a periodical local structure such as in a crystal, one can represent the susceptibility as a Fourier series, 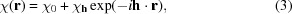
where the 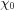 component represents the amorphous properties of the medium and 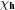 defines the local crystal structure. The reciprocal-lattice vector 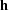 determines the family of lattice planes on which diffraction occurs.

Following the approximations in equations (2) and (3), one can derive the dispersion equation for an *s*-polarized beam: 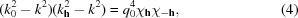
where 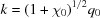 and is the wavenumber of the transmitted wave in the case of an amorphous medium. Wavenumbers 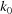 and 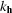 are associated with transmitted and diffracted waves in a crystal medium and differ from *k* due to the dispersion in the crystal. An explicit analytical solution of the dispersion equation in GID geometry (close to the Laue–Bragg transition point) is given by Kaganer *et al.* (1982 ▸). For simplicity, here we consider the exact vector form of the dispersion equation and solve it numerically.

The tangential component of a wavevector is constant in all media as a result of translational invariance. The difference between the vertical components of the wavevectors is expressed as the aberration coefficient ξ; these two states can then be formulated as 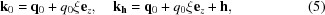
where 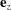 is the unit vector along the *z* axis normal to the surface of the crystal. Applying equation (5), the dispersion equation (4) becomes a polynomial equation of the fourth order with respect to ξ. Solutions of the dispersion equation (4) represent a geometrical surface – a *dispersion surface*. The Laue diffraction condition for vectors 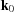 and 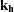 is satisfied at the intersection of the dispersion surfaces. Therefore, the shape of the dispersion surface describes the scattering process. We describe this in detail in Appendix *A* [App appa].

To calculate the amplitudes 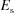 and 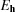 of specular reflection and diffraction waves, respectively, for a layered sample, one needs to consider the continuity conditions for the electric field (Born & Wolf, 2000 ▸) in a stratified medium. A semi-infinite vacuum is separated from a semi-infinite crystal substrate by a stack of 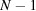 layers that have parallel interfaces. The vacuum medium has an index 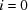 and the crystal is enumerated as 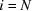. Each *i*th layer has a thickness 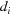. Then, applying continuity conditions to each interface one can derive 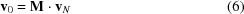
where 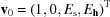 and 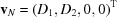 are vectors of values of the amplitudes in the vacuum and in the substrate, respectively, 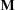 is the characteristic matrix of the sample: 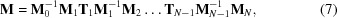
where 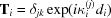 is a propagation matrix of the *i*th layer, 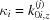 is the *j*th solution of the dispersion equation in the *i*th layer and 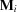 is the characteristic matrix of the *i*th layer. In the case of a mono-crystalline layer its characteristic matrix has the form
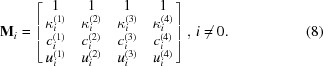
Here 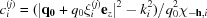 and 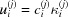. In the special case of the vacuum medium, the characteristic matrix has the form 
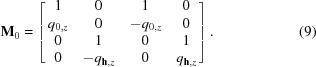
Element 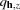 is the vertical component of the wavevector of the diffracted wave in the vacuum medium. Due to the translational invariance, the wavenumbers of the diffracted and incident waves are the same: 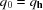. Therefore, one can derive 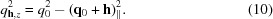
Finally, solving equation (6) with respect to 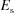 and 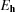 allows calculation of the amplitudes of the specular reflection and diffraction waves: 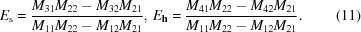



## 3. Geometry of the diffraction of evanescent X-rays   

In general, the measurement of GID rocking curves is carried out through an azimuthal rotation of the sample (rotation around the normal to the surface). We denote the angle between vector 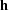 and the *xy* plane as ψ. Fig. 1 ▸ shows a typical measurement geometry for GID, where for clarity of the drawing the lattice planes are chosen to be perfectly perpendicular to the crystal surface 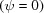, although the equations in §2 allow any orientation of the lattice planes. For 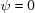 the reciprocal-lattice vector 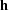 lies in the surface plane. The coordinate system is chosen such that the tangential projection of the incident wavevector lies in the plane *yz* and the *xy* plane is parallel to the surface. Thereby, the orientation of the crystal planes is described by the position of the vector 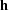, *i.e.* by azimuthal angle φ.

The Bragg condition in this geometry can be formulated as 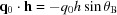, where 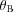 is the Bragg angle. Since lattice planes are chosen to be perpendicular to the surface, the diffracted wave propagates towards the bottom of the crystal: 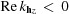. If the grazing angle of incidence is slightly above the critical angle 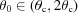 then the diffracted wave 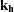 also propagates at an angle nearly parallel to the surface and reflects from the surface (Afanas’ev & Melkonyan, 1983 ▸; Cowan, 1985 ▸).

The nature of that evanescent diffracted wave with wavevector 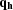 is counter-intuitive. On the one hand, that wave has a diffraction nature, but on the other hand it clearly does not satisfy the Laue diffraction condition 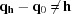, since 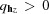. However, equation (10) holds, and that yields the interesting result that a small change of the azimuthal angle φ will lead to a much larger change in the diffraction exit angle 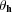. The ratio can be two to three orders of magnitude, depending on wavelength and lattice spacing.

The fact that 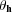 changes during azimuthal rotation is not the only consequence of equation (10). One can notice that in the case of 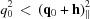 equation (10) has no solution in real numbers. In that case, by analogy with the effect of total external reflection, the evanescent diffracted wave is extinct. The point 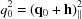 is termed the Laue point. By analogy with the situation of a critical angle, the Laue point is the azimuthal angle position below which a GID beam cannot exit the crystal.

## 4. Diffraction of an evanescent wave from a distorted subsurface layer   

In this section, we discuss predictions of the dynamical theory for the diffraction of a GID wave on a distorted crystal subsurface. The model sample is a silicon single crystal incorporating a thin ‘distorted’ single-crystalline Si layer with a lattice mismatch on top. The layer and substrate consist of the same material, and the lateral lattice mismatch of 0.1% is not sufficient to change the optical density; hence there is no optical contrast between the subsurface layer and the bulk of the single crystal. In this example, the thickness of the layer is *d* = 9 nm.

Consider the ideal case in which the Laue point is close to the Bragg condition. In that case, the crystal planes are perpendicular to the surface of the sample. As described in §3, in this case the Bragg condition is satisfied for the diffracted wave propagating towards the bottom of the crystal but due to the small angle of propagation that beam partially reflects and exits the crystal through the surface. That process is due to the diffraction that occurs in the lateral direction, and the phase rapidly changes in the vertical direction due to constraints on the wavevector in a vacuum medium [equation (10)]. That makes this scattering process sensitive to the lateral crystal structure with respect to its vertical displacement from the surface of the sample.

**Figure 2:**
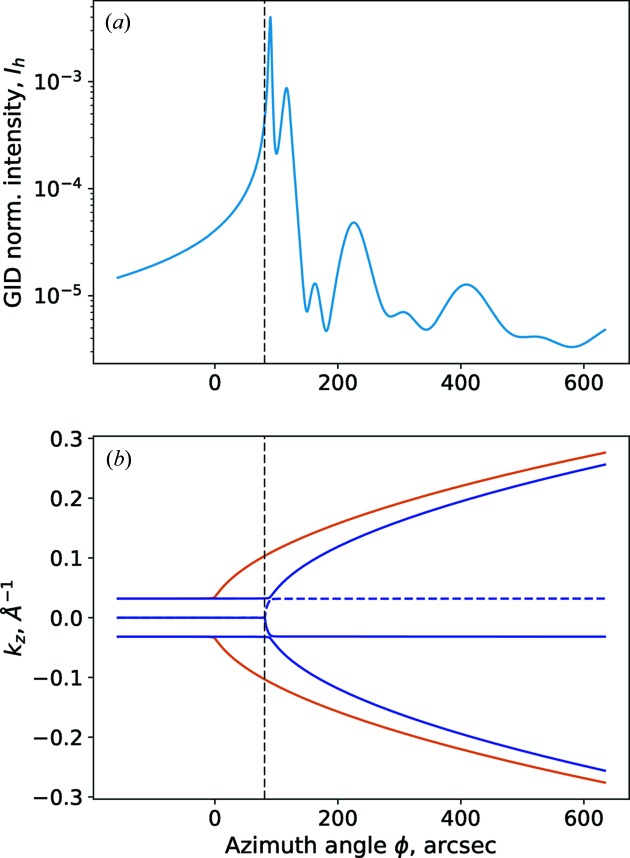
Simulation of a GID azimuthal rocking curve. Model: incident angle 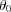 = 0.5°, wavelength λ = 0.154 nm. Si mono-crystal, surface orientation (001), scattering crystal planes series {220}, no miscut ψ = 0°; with 9 nm distorted crystalline Si layer with lateral lattice constant difference 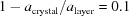. (*a*) GID azimuthal rocking curve. (*b*) Dispersion surface cross section. Orange curves – dispersion in the substrate, blue curves – dispersion in the subsurface layer, blue dashed curve – neglected root of dispersion equation.

Although the model of the sample does not have any optical contrast, there are actually strong thickness oscillations (see Fig. 2 ▸ *a*). Three details are relevant about these oscillations. First, the frequency of the oscillations varies with the azimuthal angle φ. Second, the thickness oscillations have a duplet structure: an oscillation consists of two peaks. Third, there are no oscillations for azimuthal angles bellow the Laue point. The Laue point for the layer is shown in Fig. 2 ▸ as a vertical black dashed line.

To address these features, we refer to the cross section of the dispersion surface. The curves in Fig. 2 ▸(*b*) show the dependency of the vertical component of the wavevectors with respect to the azimuthal position. Essentially, these wave­vectors describe the propagation angles of the waves in the sample. There are two branches of the dispersion surface: the orange curves are due to the waves in the substrate and the blue curves are due to the waves in the distorted layer.

During azimuthal rotation of the sample, the angles of propagation of the transmitted and reflected waves are constant. Therefore, the transmitted and reflected waves are represented by horizontal lines. Note that the dispersion curves of the transmitted and reflected waves in the layer and substrate coincide. That is due to the absence of any optical contrast. The wave reflected on the interface between the layer and substrate (black dashed line) is negligible due to the small angle of incidence and the absence of optical contrast. For this reason, that wave was neglected in the three-roots approach (see Appendix *B* [App appb]).

The dispersion curves of the diffracted waves diverge in the range above the Laue point, because the propagation angle of the diffracted wave changes with azimuthal angle, as discussed in §3. That is the reason why the frequency of the thickness oscillations shifts with azimuthal angle. The difference between orange and blue branches (see Fig. 2 ▸ *b*) yields the duplet shape of the oscillations in Fig. 2 ▸(*a*). The physical explanation for this phenomenon is the fact that there are two evanescent waves propagating through the layer representing diffraction in both the layer and in the substrate.

**Figure 3:**
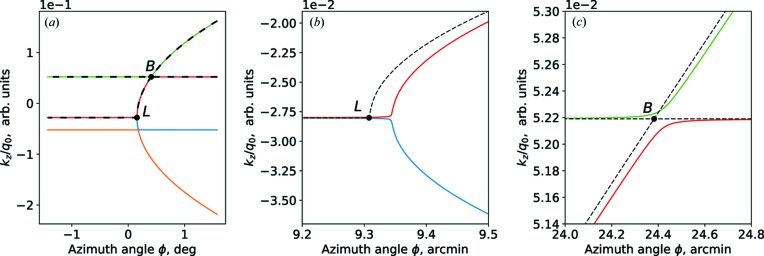
Exact (solid lines) and approximated (dashed lines) solutions of the dispersion equation. Model: incident angle 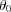 = 3°, wavelength λ = 0.154 nm. Single Si crystal, surface orientation (001), scattering crystal planes series {220}, miscut angle ψ = 2°. (*a*) Large scale, (*b*) near the Laue point, (*c*) close to the Bragg condition.

Now consider approximated analytic solutions of the dispersion equation. To derive these we will approximate the dispersion equation by a continuity condition, expressed as equation (10). Following that approach, solutions for the specular waves simply converge to translational invariance: 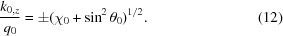
Assuming that 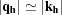 for diffracted waves we derive
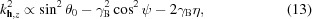
where 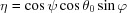 and 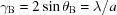 for lattice constant *a*. The approximation 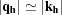 is due to the fact that in an in-plane geometry the asymmetry of the diffraction is significantly higher than the refraction. Yet the refraction can be taken into account by a factor 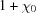 in the left-hand term of equation (10). The Laue point then satisfies the equality 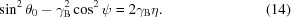
Now, consider the displacement related to the miscut angle ψ: 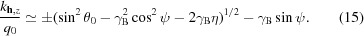
Thus, equations (15) and (12) are approximated solutions of the dispersion equation. The comparison of the approximated and exact solution is shown in Fig. 3 ▸. Therefore the diffraction is the result of coupled oscillations of these two evanescent waves.

This approximate solution can be used for easy analysis of the frequencies of the thickness oscillations, omitting complex numerical simulations. Finally, below the Laue point the dispersion curves converge to zero and remain constant. Within that range, diffracted waves in the layer are extinct and there is no interference, and thus no oscillations.

**Figure 4:**
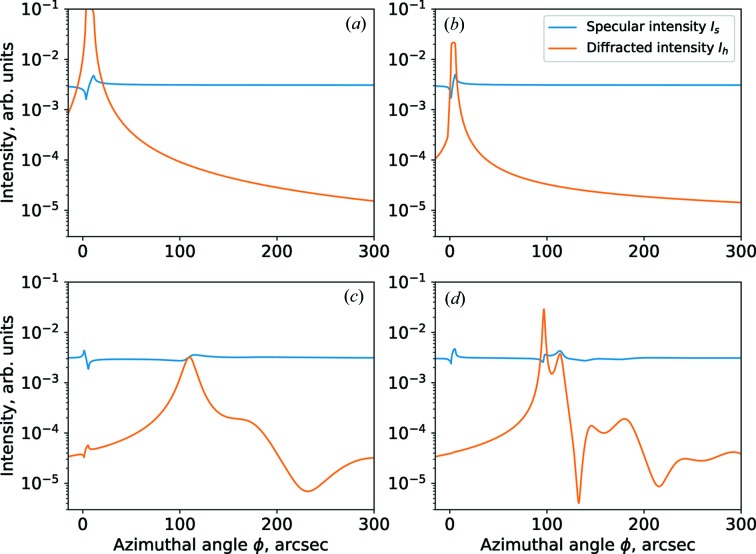
Simulations of the specular reflection intensity and GID azimuthal rocking curve. Model: Si mono-crystal with various thicknesses of the distorted layer: (*a*) *d* = 0 nm, (*b*) *d* = 2 nm, (*c*) *d* = 5 nm and (*d*) *d* = 12 nm. Incident angle 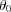 = 0.5°, wavelength λ = 0.154 nm. Single Si crystal, surface orientation (001), scattering crystal planes series {220}, miscut angle ψ = 2°.

Now, by analogy with the study of amorphous layers with specular reflection intensity (Bushuev & Oreshko, 2003 ▸), let us consider how diffraction of the evanescent wave in the distorted layer affects the specular reflection. To observe modulation of the specular reflection intensity by GID, a small miscut of ψ = 1° is considered in the model of the sample. Fig. 4 ▸ shows a simulation of the specular reflection and evanescent diffraction for various thicknesses of the top layer, as a function of the azimuthal angle around the substrate Bragg peak.

For the simulations shown in Fig. 4 ▸(*a*) there is no top layer present, and one can observe that the intensity of the specular reflection intensity is modulated by the diffraction from the substrate Bragg peak alone. With a thickness *d* = 2 nm of the top layer (Fig. 4 ▸ *b*), the diffracted intensity of the Bragg peak from the substrate decreases drastically and one can note how thickness oscillations are appearing. In Fig. 4 ▸(*c*), one can see the shape of the Bragg peak from a distorted crystal layer at a thickness of *d* = 5 nm. At this thickness, both diffraction from the subsurface and diffraction from the substrate simultaneously modulate the specular reflection. At a 12 nm layer, there is no peak of diffraction from the substrate but there is still a modulation to the specular reflection. That can be explained by the fact that there is no diffracted wave propagating towards the surface, but there is still a transmitted diffracted wave in the substrate propagating towards the bottom of the crystal.

Notice that for the case of a thick subsurface layer shown in Fig. 4 ▸(*d*), there is almost no Bragg peak from the substrate visible in the GID rocking curve. However, modulation of the specular reflection intensity by the Bragg peak from the substrate is still observed. This can be used in an experiment as a reference point in order to estimate the difference in lattice constants between the distorted layer and the substrate. The approximated solution [equation (15)] of the dispersion equation depends on the lattice constant 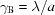 and the miscut angle ψ. The form of this equation implies that it would be possible to distinguish the influence of the lattice constant and the miscut angle on the frequencies and the shape of oscillations during the experiment.

**Figure 5:**
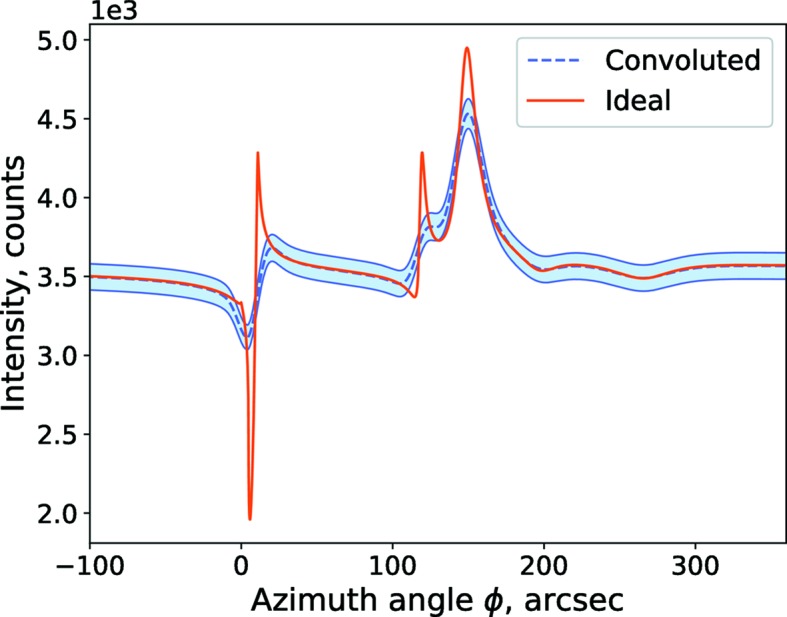
Specular reflection intensity. Comparison between ideal curve and measurement simulation. Instrument model parameters: Ge {220} monochromator, wavelength (Co *K*α-line) λ = 0.18 nm, photon energy dispersion δ*E* = 1.1 eV, incidence angle 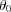 = 0.7°, angular divergence in the azimuthal direction δφ = 15 arcsec, direct beam intensity *I* = 10^4^ c.p.s., measurement time 85 min. Sample model: single Si crystal, surface orientation (001), scattering crystal planes series {220}, miscut angle ψ = 1°, with distorted *d* = 7 nm-thick layer.

Finally, in order to check the feasibility of specular reflection intensity modulation, we conducted a numerical simulation. The results of this numerical simulation are shown in Fig. 5 ▸. Here we consider the typical parameters of a laboratory X-ray instrument. For higher resolutions, compared with a conventional Cu *K*-line X-ray tube, we consider an X-ray tube with a lower photon energy: Co *K*-line. We have considered the Ge {220} monochromator. The spectral width of the Ge {220} peak for the Co *K*-line is δ*E* = 1.03 eV. For the simulation, we consider the worst parameters for which features of the ideal curve are still resolvable: photon energy dispersion δ*E* = 1.1 eV, angular divergence in the azimuthal direction δφ = 15 arcsec, direct beam intensity considered to be low *I* = 10^4^ c.p.s. due to a strong collimation of the beam. Measurement uncertainties were estimated considering that measurement took 90 min. Thus we conclude specular reflection intensity modulation measurement is feasible on a laboratory-scale X-ray diffractometer

As a logical next step, experimental evaluation of these new predictions of the dynamical theory would be necessary. In particular, the simplicity of the specular geometry and the high intensity of the reflected beam provide an opportunity to implement the technique on a relatively (compared with synchrotron) low-power laboratory-based X-ray diffractometer, as distinct from a synchrotron-based system.

## 5. Conclusions   

Implementation of the matrix formalism of the dynamical X-ray diffraction theory allowed us to describe theoretically a novel scattering process for single crystals that have defects in the crystal structure of the subsurface layers, that were introduced by the interaction with the atmosphere. GID waves induced in the subsurface interface and on the surface yield strongly asymmetric azimuthal curves. That asymmetry allowed us to estimate the difference between lattice constants of the subsurface and crystal substrate, the thickness of the distorted subsurface structure, the difference in miscut between the subsurface and substrate, and the optical contrast. Based on the obtained approximate solutions of the dispersion equation we conclude that these parameters are uncorrelated. It was also shown by means of simulations that GID modulates the specular reflection which potentially allows one to take measurements using laboratory-based instruments, making the technique widely accessible to researchers.

## Figures and Tables

**Figure 6:**
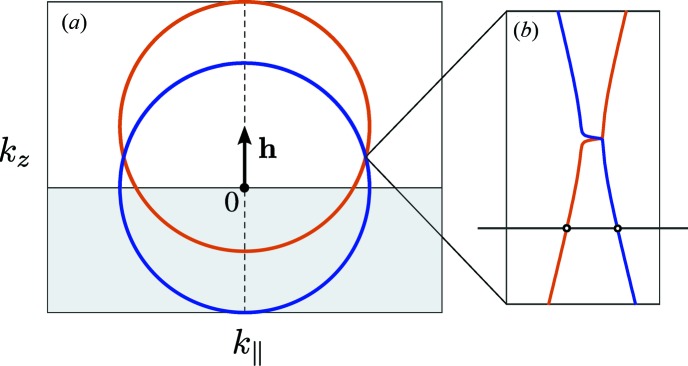
The dispersion surface cross section of symmetrical diffraction in co-planar geometry. Scattering planes: Si {111} lattice planes family. Surface orientation (111). (*a*) Large scale. (*b*) Small scale near the Bragg condition.

**Figure 7:**
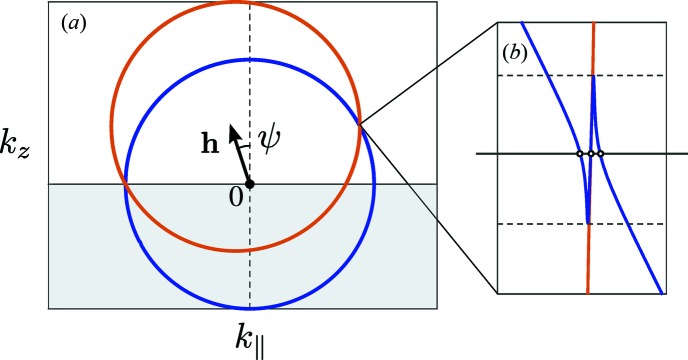
The dispersion surface cross section of asymmetrical diffraction in co-planar geometry. Scattering planes: Si {111} lattice planes family. Surface orientation (111). (*a*) Large scale. (*b*) Small scale near the Bragg condition.

## Appendix A Numerical simulation of the dispersion surface

The roots ξ of equation (4) are aberration coefficients. They determine the deviations from the vertical components of the wavevectors. Tangential components are known due to translational invariance. So, for a given set of ξ calculated for every possible incident wavevector  and using equation (5), one can calculate the dispersion surface. The physical interpretation of the roots of the dispersion equation is that each of them is assigned to a component of a Bloch wave as expressed in equation (2). Thus, one can distinguish the role of a particular component in the scattering process.

For simplicity, we first consider the trivial case of conventional symmetric X-ray diffraction. The cross section of the dispersion surface for that case is shown in Fig. 6 ▸. The axes of Fig. 6 ▸ represent the vertical and the tangential components of the wavevectors. The surface of the crystal is shown schematically in Fig. 6 ▸(*a*) as a solid black line at the centre. In the case of symmetric X-ray diffraction, the lattice planes are parallel to the surface (Fig. 6 ▸ *a*). It is similar to an Ewald construction, considering two spheres of  radius. The distance between the centres of the spheres is the length of the reciprocal-lattice vector . The sphere that has its centre in () = (0, 0) (blue circle) represents the set of  vectors while the other sphere (orange circle) represents the set of  vectors. The point of intersection corresponds to the Bragg angle.

Due to dispersion, absolute values of the incident and diffracted waves are not restricted to being the same. Indeed, close to the Bragg angle one can see deviations of the dispersion surface from a spherical shape; see Fig. 6 ▸(*b*). This is qualitatively explained by the formation of a standing wave under the Bragg conditions due to the coherence between the incident and the scattered waves. Fig. 6 ▸(*b*) shows that in the case of symmetrical diffraction there are two roots of the dispersion equation involved.

In the case in which the lattice planes are not parallel to the surface of the crystal, the process of X-ray diffraction is asymmetric. The angle of the reflected beam and the exit angle of the diffracted beam are no longer equal. The shape of the dispersion surface is different in that case (see Fig. 7 ▸). It can be observed that the Ewald sphere is rotated with reciprocal-lattice vector  at an angle ψ. In this case, part of the blue sphere (corresponding to ) to the right from the dashed line in Fig. 7 ▸(*a*) is not covered above with the orange sphere (corresponding to ). This means that an incident beam unnecessarily spawns a diffracted beam exiting the crystal. The vertical dashed line represents the Laue point. At that point, the configuration of the scattering process is changing. The diffracted beam propagates towards the bottom of the crystal instead of exiting through the surface. The effect of the Bragg–Laue transition on in-plane diffraction curves is considered in §3.

The shape of the dispersion surface close to the Bragg condition shows that three roots of the dispersion equation are involved in the diffraction (see Fig. 7 ▸ *b*). The fourth root is far on the other side of the Ewald sphere. In other words, the wave represented by this root is not coherent with the other waves; therefore, its contribution to the diffraction is negligible. We assume that in any geometry at least one wave is not coherent with the others. Following that assumption, we modify the matrix formalism to accommodate only three roots of the dispersion equation, as discussed in Appendix *B* [App appb].

## Appendix B Comparison of simulations that use three-root and four-root matrices

The propagation matrix  may become poorly conditioned due to exponents in its elements. Reducing the number of elements in the matrix  can significantly improve the calculation stability. Therefore, following our assumption (see Appendix *A* [App appa]) that only three roots of the dispersion [equation (4)] are relevant, one can reduce the matrix, neglecting the root of the incoherent wave:

For given atomic coordinates in a unit cell and atomic scattering factors for each atom in that unit cell, one can calculate the components of the susceptibility:

where *V* is the unit-cell volume,  the classical electron radius,  the coordinates of an atom in a unit cell and  the atomic scattering factor (Henke *et al.*, 1993 ▸). Summation is performed over all atoms *s* in a unit cell of the lattice.

Next, the dispersion equation [equation (4)] is solved and each root is associated with an amplitude in an array from one to four. The first root should correspond to a transmitted wave, the second to a diffracted wave towards the bottom of a crystal, the third to a specular reflection wave and the fourth to a diffracted wave towards the surface. Next, roots of the incoherent wave are neglected and then *via* equations (7) and (11) amplitudes  and  are calculated. Finally, the intensities are calculated:

We tested these approaches with three and four roots for simulation of symmetric diffraction and reflection. The discrepancies concerned the levels of machine epsilon. For the GID geometry, discrepancies were highest at a level of . However, such discrepancies are irrelevant for practical applications. Thus, three-root approximation was used for all numerical examples described in this article.
